# Testing Hardy-Weinberg Proportions in a Frequency-Matched Case-Control Genetic Association Study

**DOI:** 10.1371/journal.pone.0027642

**Published:** 2011-11-14

**Authors:** Jian Wang, Sanjay Shete

**Affiliations:** Department of Epidemiology, The University of Texas MD Anderson Cancer Center, Houston, Texas, United States of America; University Institute of Social and Preventive Medicine, Switzerland

## Abstract

In case-control genetic association studies, cases are subjects with the disease and controls are subjects without the disease. At the time of case-control data collection, information about secondary phenotypes is also collected. In addition to studies of primary diseases, there has been some interest in studying genetic variants associated with secondary phenotypes. In genetic association studies, the deviation from Hardy-Weinberg proportion (HWP) of each genetic marker is assessed as an initial quality check to identify questionable genotypes. Generally, HWP tests are performed based on the controls for the primary disease or secondary phenotype. However, when the disease or phenotype of interest is common, the controls do not represent the general population. Therefore, using only controls for testing HWP can result in a highly inflated type I error rate for the disease- and/or phenotype-associated variants. Recently, two approaches, the likelihood ratio test (LRT) approach and the mixture HWP (mHWP) exact test were proposed for testing HWP in samples from case-control studies. Here, we show that these two approaches result in inflated type I error rates and could lead to the removal from further analysis of potential causal genetic variants associated with the primary disease and/or secondary phenotype when the study of primary disease is frequency-matched on the secondary phenotype. Therefore, we proposed alternative approaches, which extend the LRT and mHWP approaches, for assessing HWP that account for frequency matching. The goal was to maintain more (possible causative) single-nucleotide polymorphisms in the sample for further analysis. Our simulation results showed that both extended approaches could control type I error probabilities. We also applied the proposed approaches to test HWP for SNPs from a genome-wide association study of lung cancer that was frequency-matched on smoking status and found that the proposed approaches can keep more genetic variants for association studies.

## Introduction

Case-control genetic association studies using unrelated individuals to find genetic variations associated with a particular disease are popular and useful. In a case-control study design, cases are those subjects with the primary disease (e.g., lung cancer, diabetes, breast cancer) and controls are those free of the primary disease. In addition to the cases and controls with respect to the primary disease, at the time of case-control collection, information about secondary phenotypes, which we define as traits associated with the primary disease of interest (i.e., predictors of the primary disease), such as smoking behavior and body mass index (BMI), are also collected. In addition to studies of primary diseases, there has been some interest in studying genetic variants associated with secondary phenotypes. Many case-control studies of primary diseases are frequency-matched on the secondary phenotypes. Frequency-matching on known risk confounders is an important and commonly used study design in case-control studies [1] to reduce the effects of confounding factors. For example, some lung cancer studies are frequency-matched on smoking behavior, as smoking is a known risk confounder for the association between lung cancer and other risk factors.

In genetic association studies, the deviation from Hardy-Weinberg proportion (HWP) of each genetic marker is typically assessed as an initial quality check procedure to identify single-nucleotide polymorphisms (SNPs) with questionable genotypes. The genetic markers that deviate from HWP are usually considered to be genotyping errors and are removed from further analysis. In general, the HWP test assumes the genotypes are sampled from the general population, and therefore, the expected genotype counts in the test should be evaluated from the general population. When the HWP test is conducted in only controls, the observed control counts are compared against the expected control counts. Recent papers [2]–[4] have shown, however, that when the disease in a case-control study is common in the general population, the controls (all of which do not have the disease) do not accurately represent the general population. Therefore, using only controls (of primary disease or secondary phenotype) for HWP testing can result in highly inflated type I error probabilities for the primary disease- and/or secondary phenotype-associated SNPs and might lead investigators to discard potential causal SNPs of the disease or secondary phenotype of interest.

Recently, new approaches have been proposed for assessing HWP in the general population for genetic case-control association studies [2]–[4]. The approaches proposed by Li and Li [2] and Yu et al [4] are based on a general likelihood ratio framework. The likelihood-based approach compares the likelihood that is maximized under the alternative hypothesis with the likelihood that is maximized under the null hypothesis (under HWP). Wang and Shete [3] proposed a mixture HWP (mHWP) exact test that uses a mixed sample of cases and controls that mimics the general population. Both the likelihood-based approach and the mHWP exact test can control the type I error rates for genetic variants associated or unassociated with the primary disease.

Both the likelihood-based and mHWP approaches will work if the study of primary disease is not frequency-matched (as shown in the Supporting Information Table S1, Table S2, Table S3 and Table S4). In this situation, individuals with the secondary phenotype are randomly sampled from among the primary disease cases and controls. However, if the case-control study is frequency-matched on the secondary phenotype, individuals with and without the secondary phenotype are not sampled randomly but on the basis of the constraints of the primary disease cases and controls. In this situation, both the likelihood-based approach and the mHWP exact test would lead to the rejection of potential causal variants associated with the primary disease and/or secondary phenotype, which would decrease the likelihood of identifying the causal or associated genetic variants in the follow-up association studies. For example, for the mHWP exact test, although the proportion of the primary disease in the mixture sample would be similar to its prevalence in the general population, the proportion of the presence of the secondary phenotype may not be consistent with the prevalence of the secondary phenotype in the general population, owing to the frequency-matching design. Thus, using the recently proposed approaches to assess HWP in the general population could introduce artificial deviations from HWP and produce inflated type I error rates for primary disease- and/or secondary phenotype-associated genetic markers.

In this article, we show that when a case-control study of primary disease was frequency-matched on the secondary phenotype, all the existing approaches failed to conserve the type I error probabilities. Therefore, we proposed alternative approaches for assessing HWP that account for frequency matching. These approaches extend the likelihood ratio test (LRT) approach [2] and the mHWP exact test [3]. We considered multiple associated and unassociated genetic variants in frequency-matched case-control studies with respect to the secondary phenotype. Simulation studies performed to investigate the performance of the proposed approaches showed that the type I error probabilities were well controlled by both extended approaches. Furthermore, we observed that, between the two approaches, the extended mHWP exact test was more likely to keep potential secondary trait and/or primary disease causal SNPs for further analysis when the secondary phenotype was more common. We also applied the proposed approaches to a real lung cancer case-control genetic association study frequency-matched on smoking behavior.

## Materials and Methods

We assumed a diallelic locus with two alleles, *A* and *a*. We denoted the three genotypes—*AA*, *Aa,* and *aa*—as a categorical random variable, *X* = (0, 1, 2). If the allele frequency of *A* is *p* and the allele frequency of *a* is (*1-p*), then the expected genotype frequencies of *AA*, *Aa,* and *aa* are *P_0_* = *p^2^*, *P_1_* = *2p(1-p),* and *P_2_* =  *(1-p)^2^*, respectively, assuming HWP in the population. We defined a binary random variable, *D* =  (0, 1), to indicate the case-control status of the primary disease, with 0 representing controls and 1 representing cases. We also defined the status of the secondary phenotype as a binary random variable, *T* =  (0, 1), with 0 representing absence of the secondary phenotype and 1 representing presence of the secondary phenotype. Let *K_ij_* denote the joint probability of secondary phenotype *T* = *i* and primary disease *D* = *j*, where *i*, *j* = 0, 1, in the general population. The prevalence of the primary disease in the general population is denoted as *f_D_*. It is easy to see that *f_D_* = *K_01_* + *K_11_*. In our studies, we assumed that the prevalence value and the joint probabilities *K_ij_* were known because usually this information can be obtained from the literature or previous studies. We assumed a case-control association study of *N* individuals, with *m* controls and *n* cases, with respect to the primary disease.

### Extended likelihood ratio test (eLRT) approach

To extend the LRT approach, we followed a strategy similar to that described by Li and Li [2]. To account for both primary disease and secondary trait associations, we considered the conditional probabilities of the genotypes given different primary disease and secondary phenotype status for the likelihood-based approach. We denoted this conditional probability as 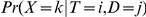 = 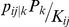, where *i*, *j* = 0, 1, *k* = 0, 1, 2, and 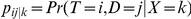 is the conditional probability that an individual is observed to have primary disease *D* = *j* and secondary phenotype *T* = *i* given the genotype *X* = *k*. Given *m* controls and *n* cases, we denoted *m_ki_* as the number of individuals in the control subjects with genotype *X* = *k* and secondary phenotype *T* = *i*, and we denoted *n_ki_* as the number of individuals in the case subjects with genotype *X* = *k* and secondary phenotype *T* = *i*. Given the sample data, the likelihood can be written as



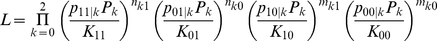
.

The data are sampled from four trinomial distributions for the genotypes, with each distribution corresponding to one of the blocks of individuals with different primary disease and secondary phenotype status; therefore, 8 parameters at most can be estimated. The above likelihood function involves 15 parameters; therefore, it is necessary to add multiple constraints to the parameters. Let 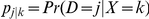, *j* = 0, 1, and *k* = 0, 1, and 2 denote the conditional probabilities of an individual with primary disease status *j* given genotype *k*, which can be written as 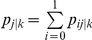. Also, we know that 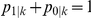 for all *k* = 0, 1, and 2. We defined the genotype relative risk for genotype *k* compared with reference genotype 0 for different scenarios: *r_ijk_* = *p_ij|k_*/*p_ij|0_* and *r_jk_* = *p_j|k_*/*p_j|0_* for *k* = 1, 2 and both *r_ij0_* and *r_j0_* equal to 1. Because we assumed that the joint probability of primary disease and secondary phenotype *K_ij_* were known in advance, the conditional probability *p_ij|k_* can be expressed using the joint probability and genotype relative risk as *p_ij|k_* = *K_ij_r_ijk_*/(*P_0_*+*r_ij1_P_1_*+*r_ij2_P_2_*), with *i*, *j* = 0, 1 and *k* = 0, 1, and 2. Similarly, because we fixed the prevalence of the primary disease, the conditional probability *p_j|k_* can be expressed as *p_j|k_* = *f_D_r_jk_*/(*P_0_*+*r_j1_P_1_*+*r_j2_P_2_*), with *j* = 0, 1 and *k* = 0, 1, and 2. The final constraint was added for the genotype frequencies, where *P_0_*+*P_1_*+*P_2_* = 1. Given these constraints for the parameters, the likelihood function can be re-written as



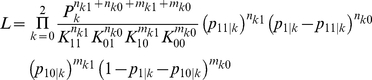
where 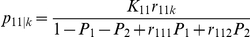, 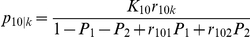, and 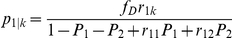 for *k* = 0, 1, and 2. We denoted 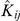, 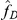, and 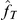 as the estimated joint probability of secondary phenotype and primary disease and the estimated prevalence values of the primary disease and secondary phenotype in the general population, respectively.

This modified likelihood function for hypothesis testing now involves 8 parameters 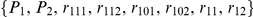. Under the null hypothesis that the genetic variant is in HWP, *P_1_* = *2p(1-p)* and *P_2_* =  *(1-p)^2^*, with *p* as the minor allele frequency (MAF). Thus, the number of parameters needing to be estimated in the likelihood function is 7 under the null hypothesis and 8 under the alternative hypothesis that the genetic variant is not in HWP. Therefore, the eLRT can be performed in a manner similar to the test proposed by Li and Li [2]. The eLRT statistic is defined as 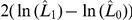, where 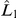 is the maximized likelihood under the alternative hypothesis and 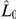 is the maximized likelihood under the null hypothesis. Asymptotically, the test statistic follows a one-degree-of-freedom chi-square distribution under the null hypothesis. To maximize the likelihood, we employed the ‘fminsearchcon’ function [5] in Matlab, which implements the simplex algorithm.

### Extended mHWP (emHWP) exact test

The basic concept of the extended mHWP (emHWP) is that, given the data of a case-control association study of primary disease frequency-matched on secondary phenotype, we try to construct a mixture sample from the data to represent the general population. In this mixture sample, the proportions of primary disease and secondary phenotype can mimic the prevalence values of primary disease and secondary phenotype in the general population, respectively.

Consider a case-control study with *N* individuals, *N* = *N_00_* + *N_10_* + *N_01_* + *N_11_*, where *N_ij_* is the number of individuals in a block of sample data with secondary trait status *i* and primary disease status *j*, where *i*, *j* = 0, 1. Let *N_m_* be the sample size of the mixture sample. To mimic the general population, the proportion of individuals in the mixture sample with secondary trait status *i* and primary disease status *j* should be consistent with the corresponding joint probability in the general population. Therefore, in the mixture sample, the number of individuals with secondary phenotype *i* and primary disease *j* should be 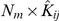 (Figure S1). For each block of individuals with secondary phenotype *i* and primary disease *j*, the number of individuals in the mixture sample must be less than the number in the original dataset. So, 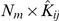 ≤ *N_ij_*, and *N_m_* ≤ min(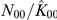,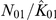,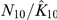,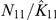). In our study, we chose *N_m_* = min(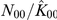,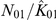,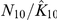,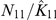) to achieve the largest possible mixture sample size and then randomly sampled 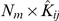 individuals from the blocks of individuals with secondary phenotype *i* and primary disease *j*. In the mixture sample, the HWP exact p value was evaluated [6]. We then employed the re-sampling procedure to obtain *M* mixture samples and assess *M* HWP exact p values, as was done in the original study by Wang and Shete [3]. The empirical distribution-based non-parametric density was constructed on the basis of *M* mixture sample p values (please see details of kernel density estimation in [3]). The maximum likelihood estimator (MLE) of this empirical distribution was obtained as the final estimate of p value for the emHWP exact test in the general population. Simulations were conducted to decide the number of mixture samples *M*, and we selected *M* = 500 in our study.

### Simulation studies

We performed simulation studies to investigate the performance of the proposed eLRT and emHWP approaches, and compared the proposed approaches to the existing approaches for HWP testing: the LRT approach proposed by Li and Li [2] and the mHWP exact test proposed by Wang and Shete [3]. We considered four independent SNPs with different associations to the primary disease and/or secondary phenotype. In addition to the genetic risk factors, we also accounted for environmental factors, including sex, ethnicity, and age, in the simulation models. The case-control status was simulated on the basis of two logistic models as follows:

Logit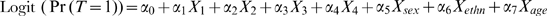,

Logit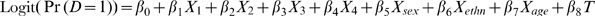.

In the two logistic models, *X_i_*, *i* = 1, …, 4, represent random variables of SNPs, and *X_sex_*, *X_ethn_*, and *X_age_* represent random variables corresponding to the environmental factors. The first logistic model was used to generate secondary phenotype status given the dataset of realizations of SNPs and environmental factors. Then, the primary disease status was generated using the second logistic model, which was conditional on the values of SNPs, environmental factors, and the secondary phenotype. We defined all the regression coefficients (*α_i_*, *i* = 1, …, 7 and *β_i_*, *i* = 1, …, 8) and prevalences of the genetic and environmental factors for the purpose of the simulation studies, as listed in Table 1. With these settings, we assumed different associations of generic variants: (1) SNP_1_ is associated with both primary disease and secondary phenotype; (2) SNP_2_ is associated with primary disease only; (3) SNP_3_ is associated with secondary phenotype only; and (4) SNP_4_ is not associated with either primary disease or secondary phenotype. The associations among all the generic variants, environmental factors, secondary phenotype, and primary disease can be represented by a network structure, as shown in Figure S2 in the Supporting Information.

**Table 1:** Parameters for simulation studies.

	Coefficients of logistic models		
Factors	*α* coefficients	*β* coefficients	Prevalence
SNP_1_	*α*_1_ = 0.4055 (OR = 1.5)	*β*_1_ = 0.4055 (OR = 1.5)	
SNP_2_	*α*_2_ = 0 (OR = 1)	*β*_2_ = 0.4055 (OR = 1.5)	
SNP_3_	*α*_3_ = 0.4055 (OR = 1.5)	*β*_3_ = 0 (OR = 1)	
SNP_4_	*α*_4_ = 0 (OR = 1)	*β*_4_ = 0 (OR = 1)	
Sex	*α*_5_ = 0.6931 (OR = 2)	*β*_5_ = 0 (OR = 1)	50% (Male)
Ethnicity	*α*_6_ = 0.4055 (OR = 1.5)	*β*_6_ = 0.4055 (OR = 1.5)	75% (Caucasian)
Age			
0-30	*α*_7_ = 0 (OR for additive model = 1)	*β*_7_ = 0.4055 (OR for additive model = 1.5)	36%
31-50			39%
Secondary Trait	NA	*β*_8_ = 1.0983 (OR = 3)	

The genotypes of the SNPs were generated with the use of the genotype frequencies assuming the SNPs were in HWP. In the simulation study, we assumed that the SNPs were common SNPs with an MAF of 40% or less common SNPs with an MAF of 10%. The values of the environmental factors were generated on the basis of their prevalence values. By using different values for the intercept coefficients *α_0_* and *β_0_*, we defined different prevalence values for the primary disease and secondary phenotype in the general population, ranging from 10% to 70%, which can represent different common diseases and common secondary traits. We did not study the scenario of a very rare disease or secondary phenotype (e.g., prevalence < 5%) because it has been shown in the previous studies [2], [3] that the standard approach for testing HWP based on controls only (with respect to the primary disease or secondary phenotype) can work well in those situations.

Given the values of the genetic variants and environmental factors, for each scenario (i.e., one pair of specific *f_D_* and *f_T_*), we generated a large amount of data on the population of interest based on the above logistic models. Therefore, the joint probability of primary disease and secondary phenotype, 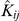, *i*, *j* = 0, 1, can be estimated from the simulated population, which would be used later in the analysis based on the proposed approaches. The case-control status was simulated assuming a dominant genetic model for all genetic variants; however, the proposed approaches are not restricted to a dominant genetic model. When a frequency-matched study based on the secondary trait is considered, the proportions of individuals with the secondary phenotype should be approximately equal in primary disease cases and controls [1]. That is, given that the secondary trait *T* is a binary random variable, the frequency-matching case-control design can be expressed using the following inequality based on conditional probabilities: | *Pr*(*T* = 1 | *D* = 0) - *Pr*(*T* = 1 | *D* = 1) | ≤ *c*, where *c* is a small fraction. We assumed the constant *c* = 0.02 in our study. Therefore, we first randomly sampled the cases of primary disease and estimated the proportion of individuals with the secondary phenotype, *Pr*(*T* = 1 | *D* = 1). According to the estimated *Pr*(*T* = 1 | *D* = 1), the proportion of individuals with the secondary phenotype in primary disease controls, *Pr*(*T* = 1 | *D* = 0), was assessed as a random number from a uniform distribution (*Pr*(*T* = 1 | *D* = 1) – *c*, *Pr*(*T* = 1 | *D* = 1) + *c*). The controls were then sampled to satisfy this estimated proportion, *Pr*(*T* = 1 | *D* = 0). In this way, we simulated 1,000,000 replicates, each with 2,000 primary disease cases and 2,000 controls frequency-matched by the secondary phenotype.

## Results

### Simulation study results

We report the observed type I error probabilities of the different approaches for testing HWP at two different significance levels for all the scenarios (i.e., the different combinations of prevalence values for the primary disease and secondary phenotype). In addition to the 0.05 nominal significance level used for candidate gene association studies, we considered the nominal significant level 0.0001, which is used as a threshold for HWP testing in genome-wide association studies [7]. All the results were evaluated based on 1,000,000 replicates. For the common SNPs (MAF = 40%), the results associated with SNP_1_, SNP_2_, SNP_3_, and SNP_4_ are reported in Tables 2, 3, 4, 5, respectively. For the less common SNPs (MAF = 10%), the results are reported in Tables 6, 7, 8, 9, respectively. Four existing approaches for testing HWP and the two proposed approaches were studied: LRT_t and mHWP_t are the LRT approach [2] and the mHWP exact test [3], respectively, which use the presence and absence of the secondary phenotype as cases and controls; LRT_d and mHWP_d use the presence and absence of the primary disease as cases and controls; the eLRT approach proposed in this article is an extension of the LRT approach proposed by Li and Li [2]; and the emHWP exact test proposed in this article is an extension of the mHWP exact test proposed by Wang and Shete [3].

**Table 2:** Estimated type I error probability for test of deviation from HWP of SNP_1_, a causal SNP to both primary disease and secondary phenotype (MAF = 40%), at 0.05 and 0.0001 significance levels in simulation studies* using different approaches for HWP testing.

Approaches	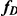	*α* = 0.05				*α* = 0.0001			
		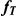				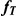			
		0.1	0.3	0.5	0.7	0.1	0.3	0.5	0.7
**LRT_t**	**0.1**	0.629540	0.575270	0.554130	0.562720	0.053358	0.041523	0.035780	0.038847
	**0.3**	0.215320	0.207040	0.206470	0.201280	0.003199	0.003054	0.002981	0.002936
	**0.5**	0.050372	0.048842	0.049259	0.049460	0.000067	0.000060	0.000111	0.000088
	**0.7**	0.210240	0.206540	0.205710	0.205040	0.003146	0.002987	0.002762	0.002758
**mHWP_t**	**0.1**	0.633310	0.584310	0.536340	0.484760	0.052694	0.027412	0.010742	0.006781
	**0.3**	0.216690	0.215840	0.205520	0.169840	0.003171	0.002029	0.000832	0.000479
	**0.5**	0.050907	0.053288	0.052970	0.046346	0.000067	0.000056	0.000050	0.000016
	**0.7**	0.212230	0.216690	0.220330	0.216660	0.003372	0.002963	0.002298	0.001488
**LRT_d**	**0.1**	0.082900	0.176400	0.179690	0.121400	0.000366	0.001921	0.002132	0.001113
	**0.3**	0.062015	0.118910	0.137840	0.106030	0.000193	0.000867	0.001054	0.000592
	**0.5**	0.056323	0.081140	0.096205	0.082865	0.000109	0.000253	0.000700	0.000413
	**0.7**	0.050907	0.059385	0.064534	0.061732	0.000212	0.000144	0.000289	0.000109
**mHWP_d**	**0.1**	0.086345	0.181400	0.184410	0.125150	0.000306	0.001731	0.001913	0.000965
	**0.3**	0.067275	0.125500	0.144800	0.112180	0.000058	0.000449	0.000555	0.000413
	**0.5**	0.054004	0.077626	0.092293	0.079165	0.000099	0.000235	0.000621	0.000393
	**0.7**	0.055775	0.064159	0.069567	0.066528	0.000116	0.000054	0.000153	0.000054
**eLRT**	**0.1**	0.050846	0.050180	0.049620	0.049718	0.000068	0.000059	0.000122	0.000038
	**0.3**	0.048648	0.050629	0.049565	0.050288	0.000102	0.000162	0.000109	0.000130
	**0.5**	0.049669	0.049533	0.049771	0.050028	0.000108	0.000089	0.000062	0.000078
	**0.7**	0.049458	0.048902	0.050458	0.049812	0.000179	0.000074	0.000111	0.000061
**emHWP**	**0.1**	0.055737	0.049401	0.037640	0.022980	0.000040	0.000006	0.000012	0.000010
	**0.3**	0.051877	0.045782	0.033138	0.020467	0.000014	0.000019	0.000021	<0.000001
	**0.5**	0.052006	0.053254	0.044670	0.029965	0.000082	0.000011	0.000008	<0.000001
	**0.7**	0.054092	0.053385	0.054638	0.054220	0.000138	0.000041	0.000107	0.000019

*Simulation studies were based on 1,000,000 replicates, each replicate with 2,000 cases in terms of primary disease and 2,000 controls frequency-matched on secondary phenotype.

MAF: minor allele frequency.

LRT_t: LRT approach, using presence and absence of secondary phenotype as cases and controls.

mHWP_t: mHWP exact test, using presence and absence of secondary phenotype as cases and controls.

LRT_d: LRT approach, using presence and absence of primary disease as cases and controls.

mHWP_d: mHWP exact test, using presence and absence of primary disease as cases and controls.

eLRT: extended LRT approach.

emHWP: extended mHWP exact test.

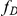
: prevalence of primary disease in general population.

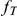
: prevalence of secondary phenotype in general population.

**Table 3:** Estimated type I error probability for test of deviation from HWP of SNP_2_, a causal SNP to primary disease but unassociated with secondary phenotype (MAF = 40%), at 0.05 and 0.0001 significance levels in simulation studies* using different approaches for HWP testing.

Approaches	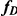	*α* = 0.05				*α* = 0.0001			
		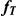				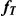			
		0.1	0.3	0.5	0.7	0.1	0.3	0.5	0.7
**LRT_t**	**0.1**	0.627260	0.573700	0.550220	0.559390	0.053796	0.040312	0.034674	0.037743
	**0.3**	0.210050	0.198150	0.200780	0.196990	0.003259	0.002515	0.002577	0.002789
	**0.5**	0.050007	0.049852	0.049675	0.051325	0.000071	0.000113	0.000062	0.000160
	**0.7**	0.216220	0.219080	0.217210	0.215410	0.003184	0.003390	0.003172	0.002850
**mHWP_t**	**0.1**	0.631460	0.584970	0.536990	0.488360	0.053770	0.026639	0.010651	0.007235
	**0.3**	0.210800	0.208130	0.200800	0.168160	0.003330	0.001737	0.000933	0.000374
	**0.5**	0.050602	0.054912	0.054477	0.048824	0.000073	0.000057	0.000016	0.000005
	**0.7**	0.218030	0.229660	0.232740	0.228650	0.003316	0.003497	0.002618	0.001366
**LRT_d**	**0.1**	0.050558	0.050690	0.050830	0.051459	0.000092	0.000117	0.000148	0.000103
	**0.3**	0.051329	0.055468	0.056119	0.052864	0.000089	0.000129	0.000150	0.000111
	**0.5**	0.050079	0.054422	0.055539	0.053985	0.000067	0.000120	0.000057	0.000145
	**0.7**	0.050483	0.051177	0.051220	0.050447	0.000094	0.000136	0.000103	0.000107
**mHWP_d**	**0.1**	0.054121	0.054870	0.054884	0.055146	0.000067	0.000117	0.000097	0.000100
	**0.3**	0.056680	0.061018	0.061881	0.057905	0.000077	0.000068	0.000089	0.000040
	**0.5**	0.048408	0.052943	0.053900	0.052227	0.000067	0.000145	0.000057	0.000132
	**0.7**	0.055468	0.056702	0.056614	0.056278	0.000024	0.000100	0.000055	0.000081
**eLRT**	**0.1**	0.050108	0.049843	0.049292	0.050494	0.000096	0.000125	0.000095	0.000046
	**0.3**	0.050273	0.050039	0.049661	0.050889	0.000065	0.000133	0.000101	0.000102
	**0.5**	0.049889	0.050504	0.049880	0.050395	0.000076	0.000065	0.000070	0.000105
	**0.7**	0.049888	0.049224	0.051004	0.050025	0.000068	0.000139	0.000087	0.000115
**emHWP**	**0.1**	0.055240	0.050562	0.038818	0.025086	0.000070	0.000032	0.000012	0.000012
	**0.3**	0.053956	0.046384	0.034750	0.022099	0.000047	0.000008	0.000012	0.000008
	**0.5**	0.052390	0.055258	0.046097	0.032279	0.000076	0.000041	0.000020	<0.000001
	**0.7**	0.055004	0.054376	0.055794	0.054795	0.000002	0.000085	0.000046	0.000102

*Simulation studies were based on 1,000,000 replicates, each replicate with 2,000 cases in terms of primary disease and 2,000 controls frequency-matched on secondary phenotype.

MAF: minor allele frequency.

LRT_t: LRT approach, using presence and absence of secondary phenotype as cases and controls.

mHWP_t: mHWP exact test, using presence and absence of secondary phenotype as cases and controls.

LRT_d: LRT approach, using presence and absence of primary disease as cases and controls.

mHWP_d: mHWP exact test, using presence and absence of primary disease as cases and controls.

eLRT: extended LRT approach.

emHWP: extended mHWP exact test.

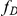
: prevalence of primary disease in general population.

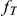
: prevalence of secondary phenotype in general population.

**Table 4:** Estimated type I error probability for test of deviation from HWP of SNP_3_, a causal SNP to secondary phenotype but unassociated with primary disease (MAF = 40%), at 0.05 and 0.0001 significance levels in simulation studies* using different approaches for HWP testing.

Approaches	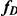	*α* = 0.05				*α* = 0.0001			
		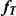				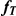			
		0.1	0.3	0.5	0.7	0.1	0.3	0.5	0.7
**LRT_t**	**0.1**	0.050237	0.049513	0.050059	0.049689	0.000102	0.000110	0.000117	0.000056
	**0.3**	0.049339	0.049678	0.049446	0.048962	0.000108	0.000116	0.000108	0.000139
	**0.5**	0.049916	0.050404	0.049220	0.049619	0.000098	0.000158	0.000111	0.000080
	**0.7**	0.049775	0.050679	0.049866	0.050453	0.000059	0.000130	0.000118	0.000171
**mHWP_t**	**0.1**	0.052723	0.053571	0.046324	0.032064	0.000102	0.000053	<0.000001	<0.000001
	**0.3**	0.050644	0.054787	0.049998	0.038074	0.000108	0.000015	0.000037	<0.000001
	**0.5**	0.050812	0.055255	0.053091	0.045548	0.000122	0.000111	0.000042	0.000004
	**0.7**	0.049322	0.053758	0.054543	0.053817	0.000059	0.000132	0.000078	0.000069
**LRT_d**	**0.1**	0.091228	0.203020	0.202380	0.132960	0.000471	0.002732	0.003004	0.001147
	**0.3**	0.072172	0.158300	0.184610	0.131600	0.000233	0.001944	0.002358	0.001177
	**0.5**	0.058820	0.103760	0.127970	0.109320	0.000192	0.000643	0.001143	0.000612
	**0.7**	0.051530	0.063688	0.073503	0.071796	0.000130	0.000196	0.000318	0.000314
**mHWP_d**	**0.1**	0.093969	0.208080	0.207090	0.136520	0.000448	0.002430	0.002673	0.001069
	**0.3**	0.077520	0.166330	0.193820	0.138970	0.000144	0.001229	0.001499	0.000816
	**0.5**	0.056407	0.099694	0.123440	0.104840	0.000180	0.000568	0.001051	0.000553
	**0.7**	0.056720	0.069087	0.078565	0.077589	0.000108	0.000100	0.000160	0.000221
**eLRT**	**0.1**	0.050286	0.049335	0.050350	0.049391	0.000137	0.000111	0.000196	0.000111
	**0.3**	0.049295	0.049636	0.049703	0.048950	0.000097	0.000118	0.000107	0.000123
	**0.5**	0.049719	0.049851	0.049578	0.049965	0.000152	0.000104	0.000117	0.000093
	**0.7**	0.049215	0.049359	0.050344	0.050839	0.000178	0.000141	0.000057	0.000179
**emHWP**	**0.1**	0.054900	0.049287	0.038968	0.023448	0.000078	0.000036	0.000020	<0.000001
	**0.3**	0.052976	0.045988	0.033970	0.019914	0.000056	<0.000001	0.000024	0.000003
	**0.5**	0.052207	0.054182	0.045483	0.030691	0.000152	0.000060	0.000022	<0.000001
	**0.7**	0.053854	0.054086	0.055279	0.055419	0.000089	0.000065	0.000033	0.000095

*Simulation studies were based on 1,000,000 replicates, each replicate with 2,000 cases in terms of primary disease and 2,000 controls frequency-matched on secondary phenotype.

MAF: minor allele frequency.

LRT_t: LRT approach, using presence and absence of secondary phenotype as cases and controls.

mHWP_t: mHWP exact test, using presence and absence of secondary phenotype as cases and controls.

LRT_d: LRT approach, using presence and absence of primary disease as cases and controls.

mHWP_d: mHWP exact test, using presence and absence of primary disease as cases and controls.

eLRT: extended LRT approach.

emHWP: extended mHWP exact test.

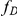
: prevalence of primary disease in general population.

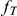
: prevalence of secondary phenotype in general population.

**Table 5:** Estimated type I error probability for test of deviation from HWP of SNP_4_, a SNP unassociated with secondary phenotype and primary disease (MAF = 40%), at 0.05 and 0.0001 significance levels in simulation studies* using different approaches for HWP testing.

Approaches	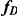	*α* = 0.05				*α* = 0.0001			
		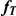				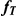			
		0.1	0.3	0.5	0.7	0.1	0.3	0.5	0.7
**LRT_t**	**0.1**	0.049895	0.051331	0.049151	0.049731	0.000081	0.000101	0.000142	0.000115
	**0.3**	0.050845	0.050014	0.048887	0.050541	0.000056	0.000080	0.000100	0.000104
	**0.5**	0.049367	0.048765	0.051168	0.049342	0.000081	0.000124	0.000131	0.000173
	**0.7**	0.050228	0.049878	0.049212	0.050788	0.000049	0.000024	0.000086	0.000070
**mHWP_t**	**0.1**	0.052873	0.056271	0.046639	0.033368	0.000089	0.000064	0.000024	<0.000001
	**0.3**	0.052488	0.055169	0.050527	0.040863	0.000060	0.000067	0.000033	0.000035
	**0.5**	0.050102	0.053512	0.055504	0.047180	0.000081	0.000106	0.000077	0.000014
	**0.7**	0.049741	0.052617	0.054543	0.054514	0.000052	0.000022	0.000065	0.000039
**LRT_d**	**0.1**	0.051678	0.052132	0.050314	0.049756	0.000103	0.000075	0.000064	0.000098
	**0.3**	0.050903	0.049942	0.048878	0.051188	0.000067	0.000130	0.000204	0.000140
	**0.5**	0.049440	0.048856	0.050964	0.049971	0.000087	0.000101	0.000135	0.000160
	**0.7**	0.050011	0.049740	0.050224	0.052106	0.000078	0.000043	0.000090	0.000070
**mHWP_d**	**0.1**	0.054502	0.055479	0.053625	0.053484	0.000093	0.000073	0.000052	0.000084
	**0.3**	0.055727	0.055445	0.054290	0.056204	0.000029	0.000059	0.000101	0.000110
	**0.5**	0.047628	0.047015	0.049317	0.047875	0.000062	0.000109	0.000135	0.000156
	**0.7**	0.055520	0.055276	0.055206	0.057024	0.000052	0.000027	0.000032	0.000055
**eLRT**	**0.1**	0.051447	0.051662	0.050660	0.049422	0.000048	0.000111	0.000135	0.000088
	**0.3**	0.050599	0.049412	0.048554	0.051584	0.000061	0.000074	0.000095	0.000073
	**0.5**	0.049071	0.048852	0.050214	0.050171	0.000082	0.000111	0.000170	0.000114
	**0.7**	0.049997	0.049947	0.049675	0.051680	0.000105	0.000076	0.000127	0.000121
**emHWP**	**0.1**	0.056339	0.052359	0.040427	0.025242	0.000017	0.000029	0.000013	0.000007
	**0.3**	0.054430	0.046910	0.034919	0.022838	0.000014	0.000025	0.000014	<0.000001
	**0.5**	0.052086	0.053611	0.046387	0.031905	0.000082	0.000072	0.000036	0.000014
	**0.7**	0.054686	0.055224	0.054902	0.056712	0.000065	0.000041	0.000034	0.000065

*Simulation studies were based on 1,000,000 replicates, each replicate with 2,000 cases in terms of primary disease and 2,000 controls frequency-matched on secondary phenotype.

MAF: minor allele frequency.

LRT_t: LRT approach, using presence and absence of secondary phenotype as cases and controls.

mHWP_t: mHWP exact test, using presence and absence of secondary phenotype as cases and controls.

LRT_d: LRT approach, using presence and absence of primary disease as cases and controls.

mHWP_d: mHWP exact test, using presence and absence of primary disease as cases and controls.

eLRT: extended LRT approach.

emHWP: extended mHWP exact test.

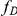
: prevalence of primary disease in general population.

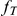
: prevalence of secondary phenotype in general population.

**Table 6:** Estimated type I error probability for test of deviation from HWP of SNP_1_, a causal SNP to both primary disease and secondary phenotype (MAF = 10%), at 0.05 and 0.0001 significance levels in simulation studies* using different approaches for HWP testing.

Approaches	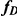	*α* = 0.05				*α* = 0.0001			
		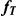				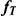			
		0.1	0.3	0.5	0.7	0.1	0.3	0.5	0.7
**LRT_t**	**0.1**	0.162010	0.153420	0.150040	0.152840	0.001597	0.001321	0.001332	0.001540
	**0.3**	0.078159	0.072453	0.071508	0.072241	0.000309	0.000347	0.000336	0.000353
	**0.5**	0.051000	0.049919	0.050700	0.049936	0.000118	0.000068	0.000063	0.000080
	**0.7**	0.071504	0.072624	0.070306	0.072665	0.000174	0.000309	0.000361	0.000241
**mHWP_t**	**0.1**	0.149180	0.135750	0.108590	0.078171	0.001100	0.000328	0.000048	0.000010
	**0.3**	0.070424	0.066110	0.056082	0.040867	0.000250	0.000092	0.000010	0.000011
	**0.5**	0.049444	0.051274	0.049291	0.040294	0.000123	0.000038	0.000022	0.000005
	**0.7**	0.076373	0.082008	0.081561	0.081048	0.000240	0.000318	0.000295	0.000101
**LRT_d**	**0.1**	0.056718	0.068336	0.067064	0.058557	0.000248	0.000260	0.000204	0.000139
	**0.3**	0.055615	0.058761	0.059443	0.056363	0.000104	0.000120	0.000239	0.000162
	**0.5**	0.051958	0.054015	0.055546	0.052000	0.000128	0.000075	0.000121	0.000079
	**0.7**	0.052333	0.052450	0.051962	0.053095	0.000102	0.000166	0.000200	0.000119
**mHWP_d**	**0.1**	0.053442	0.063423	0.062436	0.054951	0.000164	0.000132	0.000063	0.000096
	**0.3**	0.052441	0.053680	0.053728	0.051717	0.000022	0.000047	0.000054	0.000050
	**0.5**	0.045016	0.045112	0.046540	0.043593	0.000077	0.000032	0.000077	0.000079
	**0.7**	0.051805	0.051895	0.050645	0.051822	0.000053	0.000086	0.000084	0.000052
**eLRT**	**0.1**	0.052115	0.052281	0.054407	0.075478	0.000311	0.000155	0.000511	0.001738
	**0.3**	0.052444	0.049599	0.052305	0.059940	0.000113	0.000149	0.000202	0.000639
	**0.5**	0.051163	0.049736	0.050396	0.056037	0.000155	0.000060	0.000073	0.000300
	**0.7**	0.054020	0.052456	0.051402	0.058749	0.000111	0.000155	0.000187	0.000327
**emHWP**	**0.1**	0.049613	0.040647	0.027971	0.016156	0.000065	0.000012	0.000013	<0.000001
	**0.3**	0.048224	0.035411	0.024398	0.013660	0.000016	0.000002	<0.000001	<0.000001
	**0.5**	0.050066	0.048976	0.038241	0.024400	0.000115	0.000024	<0.000001	<0.000001
	**0.7**	0.052258	0.051763	0.049974	0.051433	0.000071	0.000102	0.000107	0.000059

*Simulation studies were based on 1,000,000 replicates, each replicate with 2,000 cases in terms of primary disease and 2,000 controls frequency-matched on secondary phenotype.

MAF: minor allele frequency.

LRT_t: LRT approach, using presence and absence of secondary phenotype as cases and controls.

mHWP_t: mHWP exact test, using presence and absence of secondary phenotype as cases and controls.

LRT_d: LRT approach, using presence and absence of primary disease as cases and controls.

mHWP_d: mHWP exact test, using presence and absence of primary disease as cases and controls.

eLRT: extended LRT approach.

emHWP: extended mHWP exact test.

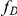
: prevalence of primary disease in general population.

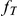
: prevalence of secondary phenotype in general population.

**Table 7:** Estimated type I error probability for test of deviation from HWP of SNP_2_, a causal SNP to primary disease but unassociated with secondary phenotype (MAF = 10%), at 0.05 and 0.0001 significance levels in simulation studies* using different approaches for HWP testing.

Approaches	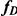	*α* = 0.05				*α* = 0.0001			
		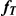				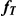			
		0.1	0.3	0.5	0.7	0.1	0.3	0.5	0.7
**LRT_t**	**0.1**	0.161340	0.154220	0.148260	0.148780	0.001358	0.001472	0.001255	0.001499
	**0.3**	0.076030	0.074891	0.074848	0.073556	0.000307	0.000159	0.000388	0.000311
	**0.5**	0.051226	0.050695	0.049906	0.051283	0.000158	0.000184	0.000166	0.000090
	**0.7**	0.069704	0.071344	0.067986	0.070402	0.000182	0.000189	0.000176	0.000303
**mHWP_t**	**0.1**	0.146860	0.141050	0.117140	0.085517	0.000991	0.000629	0.000140	0.000036
	**0.3**	0.066561	0.068085	0.061517	0.045970	0.000224	0.000075	0.000052	0.000000
	**0.5**	0.048454	0.051398	0.049869	0.044307	0.000104	0.000113	0.000036	0.000026
	**0.7**	0.073637	0.079299	0.077750	0.078682	0.000257	0.000214	0.000161	0.000201
**LRT_d**	**0.1**	0.052950	0.050386	0.050286	0.049250	0.000114	0.000102	0.000200	0.000075
	**0.3**	0.050115	0.050408	0.050887	0.050550	0.000118	0.000105	0.000078	0.000099
	**0.5**	0.051150	0.050612	0.051208	0.050205	0.000119	0.000088	0.000198	0.000122
	**0.7**	0.051033	0.051331	0.049037	0.050975	0.000095	0.000080	0.000077	0.000127
**mHWP_d**	**0.1**	0.052819	0.049958	0.050091	0.048436	0.000069	0.000092	0.000117	0.000029
	**0.3**	0.050449	0.051010	0.052295	0.051333	0.000013	0.000060	0.000025	0.000030
	**0.5**	0.047014	0.046632	0.046919	0.046702	0.000088	0.000086	0.000134	0.000111
	**0.7**	0.052191	0.051853	0.050738	0.052515	0.000054	0.000060	0.000059	0.000088
**eLRT**	**0.1**	0.051798	0.050953	0.053997	0.060978	0.000122	0.000115	0.000093	0.001070
	**0.3**	0.049308	0.051280	0.052182	0.057070	0.000130	0.000166	0.000082	0.000243
	**0.5**	0.051033	0.050921	0.050215	0.052377	0.000125	0.000134	0.000189	0.000223
	**0.7**	0.052914	0.051716	0.049788	0.054130	0.000081	0.000079	0.000095	0.000312
**emHWP**	**0.1**	0.051345	0.043123	0.033298	0.018485	0.000030	0.000009	0.000007	<0.000001
	**0.3**	0.046360	0.039182	0.029185	0.016736	0.000023	0.000018	<0.000001	<0.000001
	**0.5**	0.050293	0.050347	0.040255	0.027978	0.000091	0.000057	<0.000001	0.000041
	**0.7**	0.051471	0.050799	0.049526	0.051332	0.000034	0.000060	0.000051	0.000072

*Simulation studies were based on 1,000,000 replicates, each replicate with 2,000 cases in terms of primary disease and 2,000 controls frequency-matched on secondary phenotype.

MAF: minor allele frequency.

LRT_t: LRT approach, using presence and absence of secondary phenotype as cases and controls.

mHWP_t: mHWP exact test, using presence and absence of secondary phenotype as cases and controls.

LRT_d: LRT approach, using presence and absence of primary disease as cases and controls.

mHWP_d: mHWP exact test, using presence and absence of primary disease as cases and controls.

eLRT: extended LRT approach.

emHWP: extended mHWP exact test.

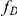
: prevalence of primary disease in general population.

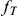
: prevalence of secondary phenotype in general population.

**Table 8:** Estimated type I error probability for test of deviation from HWP of SNP_3_, a causal SNP to secondary phenotype but unassociated with primary disease (MAF = 10%), at 0.05 and 0.0001 significance levels in simulation studies* using different approaches for HWP testing.

Approaches	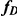	*α* = 0.05				*α* = 0.0001			
		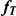				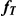			
		0.1	0.3	0.5	0.7	0.1	0.3	0.5	0.7
**LRT_t**	**0.1**	0.050153	0.050133	0.050911	0.052248	0.000080	0.000135	0.000112	0.000056
	**0.3**	0.049309	0.050903	0.049561	0.051465	0.000120	0.000082	0.000117	0.000119
	**0.5**	0.051165	0.049958	0.050187	0.051265	0.000108	0.000064	0.000067	0.000091
	**0.7**	0.050114	0.050480	0.051380	0.050936	0.000130	0.000077	0.000128	0.000052
**mHWP_t**	**0.1**	0.050368	0.048035	0.037602	0.024191	0.000043	0.000019	0.000016	0.000013
	**0.3**	0.048566	0.050854	0.042486	0.031576	0.000099	0.000009	<0.000001	0.000002
	**0.5**	0.049050	0.050853	0.048492	0.041053	0.000122	0.000040	0.000034	0.000007
	**0.7**	0.046458	0.049772	0.051727	0.049122	0.000094	0.000052	0.000084	0.000010
**LRT_d**	**0.1**	0.058316	0.073940	0.072316	0.061562	0.000135	0.000253	0.000289	0.000271
	**0.3**	0.054009	0.067947	0.066766	0.060957	0.000112	0.000250	0.000186	0.000199
	**0.5**	0.053243	0.058409	0.061204	0.057918	0.000116	0.000142	0.000220	0.000099
	**0.7**	0.051326	0.053231	0.054928	0.053418	0.000124	0.000075	0.000089	0.000086
**mHWP_d**	**0.1**	0.054332	0.068308	0.066194	0.057182	0.000055	0.000132	0.000114	0.000135
	**0.3**	0.051343	0.062151	0.061383	0.056378	0.000032	0.000063	0.000031	0.000086
	**0.5**	0.045976	0.048570	0.050252	0.047917	0.000066	0.000104	0.000110	0.000072
	**0.7**	0.050590	0.051410	0.053220	0.051586	0.000039	0.000015	0.000039	0.000050
**eLRT**	**0.1**	0.051076	0.050677	0.054545	0.057184	0.000142	0.000101	0.000154	0.000071
	**0.3**	0.049723	0.050855	0.051065	0.055910	0.000178	0.000066	0.000060	0.000084
	**0.5**	0.051668	0.050298	0.050811	0.054281	0.000129	0.000053	0.000117	0.000073
	**0.7**	0.051233	0.051140	0.051973	0.056249	0.000035	0.000061	0.000173	0.000159
**emHWP**	**0.1**	0.049861	0.040333	0.028715	0.015904	0.000039	0.000021	0.000006	<0.000001
	**0.3**	0.047309	0.038727	0.026424	0.014201	0.000034	0.000002	<0.000001	<0.000001
	**0.5**	0.050091	0.048795	0.039083	0.025750	0.000115	0.000026	0.000025	<0.000001
	**0.7**	0.050428	0.051245	0.051873	0.051315	0.000025	0.000028	0.000076	0.000043

*Simulation studies were based on 1,000,000 replicates, each replicate with 2,000 cases in terms of primary disease and 2,000 controls frequency-matched on secondary phenotype.

MAF: minor allele frequency.

LRT_t: LRT approach, using presence and absence of secondary phenotype as cases and controls.

mHWP_t: mHWP exact test, using presence and absence of secondary phenotype as cases and controls.

LRT_d: LRT approach, using presence and absence of primary disease as cases and controls.

mHWP_d: mHWP exact test, using presence and absence of primary disease as cases and controls.

eLRT: extended LRT approach.

emHWP: extended mHWP exact test.

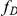
: prevalence of primary disease in general population.

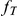
: prevalence of secondary phenotype in general population.

**Table 9:** Estimated type I error probability for test of deviation from HWP of SNP_4_, a SNP unassociated with secondary phenotype and primary disease (MAF = 10%), at 0.05 and 0.0001 significance levels in simulation studies* using different approaches for HWP testing.

Approaches	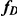	*α* = 0.05				*α* = 0.0001			
		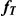				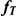			
		0.1	0.3	0.5	0.7	0.1	0.3	0.5	0.7
**LRT_t**	**0.1**	0.049955	0.050560	0.050754	0.051529	0.000103	0.000092	0.000086	0.000119
	**0.3**	0.051255	0.050142	0.050185	0.050944	0.000117	0.000074	0.000141	0.000090
	**0.5**	0.050236	0.050098	0.050188	0.051949	0.000147	0.000081	0.000078	0.000095
	**0.7**	0.050851	0.050228	0.050880	0.051135	0.000037	0.000165	0.000099	0.000092
**mHWP_t**	**0.1**	0.048855	0.050711	0.042215	0.028967	0.000066	0.000045	0.000006	<0.000001
	**0.3**	0.049976	0.050795	0.046092	0.035414	0.000089	0.000044	0.000016	0.000009
	**0.5**	0.047727	0.050971	0.049961	0.043757	0.000117	0.000043	0.000008	<0.000001
	**0.7**	0.045951	0.049392	0.051668	0.050732	0.000025	0.000169	0.000070	0.000046
**LRT_d**	**0.1**	0.051172	0.049296	0.050029	0.050813	0.000096	0.000145	0.000066	0.000052
	**0.3**	0.052033	0.050256	0.050089	0.050230	0.000070	0.000084	0.000031	0.000142
	**0.5**	0.051063	0.049871	0.049610	0.051423	0.000151	0.000073	0.000099	0.000086
	**0.7**	0.051395	0.050905	0.051639	0.050943	0.000083	0.000135	0.000115	0.000121
**mHWP_d**	**0.1**	0.050153	0.047861	0.048359	0.049300	0.000076	0.000108	0.000074	0.000052
	**0.3**	0.051980	0.050553	0.050566	0.050233	0.000033	0.000014	0.000026	0.000069
	**0.5**	0.045581	0.044546	0.044106	0.045819	0.000101	0.000062	0.000115	0.000065
	**0.7**	0.051886	0.050960	0.051851	0.051027	0.000031	0.000083	0.000074	0.000079
**eLRT**	**0.1**	0.051528	0.051132	0.052837	0.055981	0.000132	0.000197	0.000075	0.000086
	**0.3**	0.052157	0.049806	0.051373	0.053434	0.000089	0.000055	0.000124	0.000068
	**0.5**	0.051175	0.050076	0.049524	0.052920	0.000138	0.000089	0.000126	0.000059
	**0.7**	0.051641	0.051034	0.052126	0.051752	0.000098	0.000104	0.000099	0.000110
**emHWP**	**0.1**	0.050713	0.044745	0.032836	0.020672	0.000013	0.000041	<0.000001	0.000009
	**0.3**	0.050183	0.041807	0.029977	0.017740	0.000007	0.000016	<0.000001	<0.000001
	**0.5**	0.049308	0.049678	0.039879	0.029402	0.000125	0.000048	0.000036	<0.000001
	**0.7**	0.051104	0.050868	0.052230	0.051118	0.000030	0.000077	0.000064	0.000061

*Simulation studies were based on 1,000,000 replicates, each replicate with 2,000 cases in terms of primary disease and 2,000 controls frequency-matched on secondary phenotype.

MAF: minor allele frequency.

LRT_t: LRT approach, using presence and absence of secondary phenotype as cases and controls.

mHWP_t: mHWP exact test, using presence and absence of secondary phenotype as cases and controls.

LRT_d: LRT approach, using presence and absence of primary disease as cases and controls.

mHWP_d: mHWP exact test, using presence and absence of primary disease as cases and controls.

eLRT: extended LRT approach.

emHWP: extended mHWP exact test.

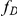
: prevalence of primary disease in general population.

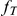
: prevalence of secondary phenotype in general population.

Table 2 reports the type I error probabilities of different approaches for testing HWP for SNP_1_ (MAF = 40%) at 0.05 and 0.0001 significance levels. SNP_1_ was associated with both the primary disease and the secondary phenotype in the simulations. The LRT approach and the mHWP exact test using individuals with presence and absence of the secondary phenotype as cases and controls (LRT_t and mHWP_t) provided similar type I error rates, and neither could control the type I error rate in most of the scenarios. Both approaches also performed very similarly when using individuals with presence and absence of the primary disease as cases and controls (LRT_d and mHWP_d); both could control the type I error rate in more scenarios than LRT_t and mHWP_t but still resulted in an inflated type I error rate in many scenarios. Finally, the newly proposed eLRT approach and emHWP exact test both controlled the type I error rate well. For example, when prevalence values of both the primary disease and secondary phenotype were 0.3, given a 0.05 significance level, the type I error rates of the LRT_t, mHWP_t, LRT_d, and mHWP_d approaches were 0.207040, 0.215840, 0.118910, and 0.125500, respectively, which were all highly inflated; the type I error rates of the eLRT and emHWP approaches were 0.050629 and 0.045782, respectively, which agreed very well with the nominal significance value of 0.05. When the nominal significance level was 0.0001 and both *f_D_* and *f_T_* were set as 0.3, we observed a similar trend in type I errors: the type I error rates of the existing approaches were 0.003054, 0.002029, 0.000867 and 0.000449, respectively, which were highly inflated, whereas the type I error rates of the eLRT and emHWP approaches were 0.000162 and 0.000019, respectively, which agreed very well with the nominal significance value of 0.0001.

When the genetic variant was only associated with the primary disease (SNP_2_, Table 3), the LRT approach and the mHWP exact test using individuals with presence and absence of primary disease as cases and controls (LRT_d and mHWP_d) could conserve the type I error rates for all the scenarios. This was expected because SNP_2_ was associated with the primary disease only, and this assumption was the focus in the original studies of these two approaches [2], [3]. However, LRT_t and mHWP_t led to inflated type I error rates. And again, we observed that the type I error rates were well controlled by both of the proposed approaches, eLRT and the emHWP exact test.

 When the genetic variant was only associated with the secondary phenotype (SNP_3_, Table 4), it was not surprising to see that the LRT approach and the mHWP exact test using individuals with presence and absence of the secondary phenotype as cases and controls (LRT_t and mHWP_t) could control the type I error rates in all scenarios. However, LRT_d and mHWP_d led to inflated type I error rates in many situations. As in the results for SNP_1_ and SNP_2_, both the proposed approaches (eLRT and emHWP) still controlled type I error rates well for all scenarios.

Last, when the genetic variant was not associated with the primary disease or the secondary phenotype (SNP_4_, Table 5), all of the approaches controlled the type I error rates well for all scenarios.

Therefore, the results reported in Tables 2, 3, 4, 5 for the common SNPs (MAF = 40%) show that the proposed eLRT and emHWP approaches could control the type I error rates for all SNPs with different types of associations with primary or secondary phenotypes and all scenarios with different prevalence values. It also should be noted that when the primary disease was less common (e.g., 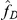 =  0.1 ∼ 0.5) and the secondary phenotype was very common (e.g., 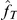 =  0.5 ∼ 0.7), the eLRT approach tended to have a larger type I error rate than the emHWP exact test, which means that the emHWP exact test is more likely to keep the promising genetic variants than the eLRT approach in these situations. It is possible that actual studies of primary disease and secondary phenotype could fall within these ranges of prevalence values. For example, in a study of overweight based on data collected for studying type 2 diabetes, the prevalence of type 2 diabetes (primary disease) was about 10% in the U.S. [8] and the prevalence of overweight was about 66% in the U.S. [9]. In this situation, the emHWP test would be preferable to the eLRT approach. At a very low nominal significance level (0.0001), the eLRT, but not the emHWP, approach had a slightly inflated type I error rate. Thus, the emHWP exact test is also more likely to keep the promising genetic variants than the eLRT approach at a low nominal significance level.

When the SNPs of interest were less common (MAF = 10%, Tables 6, 7, 8, 9), we observed similar trends in the results for all SNPs with different associations. As expected, the inflation in type I error rates of the existing approaches was not as significant as that for common SNPs (MAF = 40%, Tables 2, 3, 4, 5). Especially, we noticed that the LRT_d and mHWP_d approaches could conserve the type I error better in many, but not all, situations. For example, for SNP_1_ (Table 6, associated with both the primary disease and secondary phenotype), when the prevalence values of both primary disease and secondary phenotype were 0.3, given a 0.05 significance level, the type I error rates of the different existing approaches were 0.072453, 0.066110, 0.058761, and 0.053680, respectively, whereas the type I error rates of the proposed approaches were 0.049599 and 0.035411, respectively, which were well-controlled at the 0.05 level. The performance of the emHWP exact test for the less common SNPs was very similar to that for the common SNPs at both the 0.05 and 0.0001 significance levels for all scenarios. However, the eLRT approach had inflated type I error rates at the 0.0001 level of significance in some situations for the less common SNPs. This observation further suggested that the emHWP exact test is more favorable than the eLRT approach in these situations.

Although the previously proposed LRT approach and mHWP exact test would work for certain SNPs in some scenarios, in reality, the HWP tests are performed before the association tests. Therefore, one would not know the underlying real associations of SNPs with the primary disease and/or secondary phenotype when performing the HWP tests, and the existing approaches might lead to the removal of genetic variants potentially associated with the primary disease and/or secondary phenotype. In contrast, the proposed emHWP test performed uniformly well at controlling the type I error rates for all four SNPs with different associations to the primary disease and secondary phenotype in all scenarios.

 We also conducted simulation studies to evaluate the performances of all the approaches to HWP testing for the unmatched case-control study of primary disease and reported the type I error results in Supporting Information Tables S1, S2, S3, S4. We considered common SNPs with MAF = 40% and defined different prevalence values for primary disease and secondary phenotype in the general population, ranging from 10% to 90%. The type I errors were evaluated at a nominal significance level of 0.05. All the results were based on 1,000 replicates, each with 1,000 primary disease cases and 1,000 randomly sampled controls. As in the frequency-matched case-control studies, the proposed eLRT approach and the emHWP exact test were both able to control the type I error rates for all SNPs and all scenarios in the unmatched case-control studies. Therefore, the proposed eLRT approach and emHWP exact test are robust for different study designs. In addition, the LRT approach and the mHWP exact test using individuals with presence and absence of primary disease as cases and controls also performed well for all SNPs and all scenarios, as expected.

### Application to lung cancer data

To examine the performance of the proposed eLRT and emHWP approaches, we also applied them to the case-control study of lung cancer frequency-matched on smoking status. This analysis included 2,291 individuals, with 1,154 lung cancer patients and 1,137 controls frequency-matched to the cases by age, sex, and smoking status [10]. The data were collected for a case-control study of lung cancer. All the case and control subjects were ever smokers: 1,260 former smokers and 1,031 current smokers. All the individuals were Caucasian. Lung cancer cases were accrued at The University of Texas MD Anderson Cancer Center and were histologically confirmed. Controls were ascertained through a multi-specialty physician practice from the same area. Questionnaire data were obtained by personal interview in the original study. This study was approved by the institutional review board at MD Anderson Cancer Center, and all participants provided written informed consent (LAB10-0347). In the lung cancer genome-wide association study, 317,498 tagging SNPs were genotyped [11]. We only included the autosomal SNPs in this study. We further excluded the SNPs with MAF < 0.05, and therefore, 300,738 SNPs were left in the analysis.

 We were interested in determining how many SNPs would be rejected in the quality check procedure using the different approaches for testing HWP. From the simulation studies, we found that the LRT approach and mHWP exact test performed very similarly; therefore, we only reported the results obtained using the LRT approach with either (1) the presence and absence of lung cancer as cases and controls (LRT_d) or (2) current and former smokers as cases and controls (LRT_t). To evaluate eLRT and emHWP, we first obtained the prevalence values of lung cancer 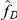 and current smokers 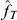 in ever smokers from the literature (0.14 and 0.498, respectively) [12], [13]. We then estimated the conditional probability of lung cancer cases given current smokers in the ever smokers as 0.2545 [12]. Therefore, we could calculate the estimated joint probabilities of lung cancer and smoking status 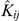, where *i*, *j* = 0, 1, with 1 representing lung cancer patients and current smokers and 0 representing lung cancer-free controls and former smokers. For example, 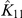 =  0.2545 × 0.14 = 0.0356 and 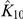 = 0.14 – 0.0356 = 0.1044. 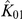 and 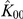 can then be calculated accordingly. Table 10 reports the numbers of SNPs that would be rejected and removed in the quality check procedure using the different HWP testing approaches, including LRT_d, LRT_t, eLRT, and emHWP, at different commonly used nominal significance levels (from 0.005 to 0.000001) in genome-wide association studies. We observed that for all significance levels, the proposed eLRT and emHWP approaches always rejected fewer SNPs than the LRT approach. The emHWP approach always rejected the fewest SNPs, whereas LRT_t always rejected the most SNPs, among all four approaches. For example, when the nominal significance level was 0.0001, 1,121 and 812 SNPs would be rejected and removed by using the LRT_t and LRT_d, respectively, whereas only 798 and 637 SNPs would be rejected and removed by using the proposed eLRT and emHWP approaches, respectively. Compared with the LRT_t approach, the emHWP approach would keep 484 more SNPs for further analysis of the association of lung cancer and/or smoking status.

**Table 10:** Numbers of SNPs rejected using different approaches for testing HWP in the case-control genetic association study of lung cancer frequency-matched on smoking behavior.

	Number of rejections			
Significance	LRT_t	LRT_d	eLRT	emHWP
**0.005**	3445	3049	2949	2320
**0.001**	1778	1405	1374	1057
**0.0005**	1501	1130	1116	847
**0.0001**	1121	812	798	637
**0.00005**	1031	743	734	580
**0.00001**	891	617	608	478
**0.000005**	825	568	568	453
**0.000001**	730	502	490	419

## Discussion

In this article, we propose two new approaches (eLRT and emHWP) for testing HWP in genetic association studies in which the primary disease cases and controls are frequency-matched on the secondary phenotype. These two approaches are extensions of two recently proposed approaches, the LRT approach [2] and the mHWP exact test [3], that further account for the frequency-matching design with respect to the secondary phenotype. When the case-control study of primary disease is frequency-matched based on the secondary phenotype, which is correlated with the primary disease, statistically speaking, it can be considered to analyze one phenotype with four possible categories, and the likelihood function in the eLRT approach was constructed under this scenario. Similar thinking could be applied to the development of the emHWP exact test. Moreover, the approaches proposed can be extended to obtain estimates and standard errors of the allele frequency. The performance of the two approaches was investigated via simulation studies, as well as an analysis of an association study of lung cancer frequency-matched on smoking status.

We compared the proposed approaches to the existing LRT approach and mHWP exact test. On the basis of the results of our simulation studies, when the study of primary disease was frequency-matched on the secondary phenotype, the existing LRT and mHWP exact test provided inflated type I error rates for many scenarios for the different SNPs. In contrast, the newly proposed emHWP approach uniformly and effectively controlled the type I error probability in all scenarios examined for different SNPs associated with the secondary phenotype and/or the primary disease. For some scenarios (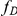 is small while 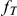
is large), the emHWP approach is more likely than the eLRT approach to keep SNPs associated with the primary disease and/or secondary phenotype in the analysis. The performance of the emHWP for less common SNPs (MAF = 10%) is similar to that for common SNPs at different significance levels for all the SNPs. The eLRT approach, on the other hand, behaved slightly differently at a low significance level when the SNPs were less common. It tends to provide inflated type I errors at a low significance level, especially when the disease is less common but the secondary phenotype is very common (i.e., *f_D_* = 0.1 and *f_T_* = 0.7) and the SNPs are associated with the primary disease. Therefore, we recommend the emHWP exact test as a better HWP test that has a greater chance of keeping potentially associated SNPs for future association analysis.

In reality, the prevalence values of the primary disease and secondary phenotype, as well as their joint distribution, cannot be known for certain. Therefore, we also evaluated the robustness of the proposed approaches to the estimated prevalence values and joint distribution using simulations. We considered a range of primary disease prevalence and conditional probabilities centered on the true prevalence and true conditional probabilities: [*f_D_* – *Δ_D_*, *f_D_* + *Δ_D_*], [*f_T_* in cases – *Δ_1_*, *f_T_* in cases + *Δ_1_*] and [*f_T_* in controls – *Δ_0_*, *f_T_* in controls + *Δ_0_*]. The error terms were defined as 20% of the true values, for example *Δ_D_* = 20% × *f_D_*. The miss-specified secondary phenotype prevalence value and joint probabilities can be evaluated by using the primary disease prevalence and conditional probabilities defined above. We found that all the results were very similar to those obtained using the real prevalence values and joint distribution. Therefore, the misspecification of prevalence values and joint distribution will not inflate the type I error rate of the proposed approaches (as in the previous work [3]). The interactive effects of secondary phenotype and genetic variants on primary disease might have some impact on the test for deviation from HWP for genetic variants, which could be an interesting topic for future study.

In addition to the simulation studies, we also applied the eLRT and emHWP approaches to a real case-control genetic association study of lung cancer frequency-matched on smoking status and compared the numbers of rejected SNPs to those obtained using the LRT approach in the quality check procedure. In the original lung cancer study, the lung cancer controls were frequency-matched by smoking status to the cases. The proposed approaches always rejected, and thus removed, fewer SNPs than the LRT approach. We are not claiming that the SNPs kept using our approaches are causal or associated with the primary disease or secondary phenotype, as such claims would require validation by independent studies as dictated by the genome-wide association study guidelines. Our main goal is to increase the likelihood of not filtering potentially associated SNPs in the data cleanup stage. In other words, the eLRT and emHWP approaches have a higher likelihood of keeping SNPs for further association analysis, and the additional SNPs kept could potentially be associated with either the secondary phenotype or primary disease, according to our simulation results.

To summarize, in this article, we extended the recently proposed HWP testing approaches, the LRT approach and mHWP exact test, to frequency-matched case-control study. We showed that when the study of the primary disease is unmatched, the proposed eLRT and emHWP approaches are robust and provide results similar to those obtained with the existing approaches; when the study of primary disease is frequency-matched with respect to the secondary phenotype, the proposed approaches are better HWP tests than the existing approaches. For frequency-matched studies based on the secondary phenotype, the eLRT and emHWP approaches will improve our ability to keep SNPs potentially associated with the secondary phenotype and/or the primary disease.

## Supporting Information

Figure S1 Construction of a mixture sample from the dataset of a case-control study of primary disease.
(DOC)

Figure S2 Network structure representing associations among genetic variants, environmental factors, secondary phenotype, and primary disease.
(DOC)

Table S1 Estimated type I error probability for test of deviation from HWP of SNP_1_, a SNP causal to both primary disease and secondary phenotype (MAF = 40%), at a 0.05 significance level in simulation studies using different approaches for HWP testing.
(DOC)

Table S2 Estimated type I error probability for test of deviation from HWP of SNP_2_, a causal SNP to primary disease but unassociated with secondary phenotype (MAF = 40%), at a 0.05 significance level in simulation studies using different approaches for HWP testing.
(DOC)

Table S3 Estimated type I error probability for test of deviation from HWP of SNP_3_, a causal SNP to secondary phenotype but unassociated with primary disease (MAF = 40%), at a 0.05 significance level in simulation studies using different approaches for HWP testing.
(DOC)

Table S4 Estimated type I error probability for test of deviation from HWP of SNP_4_, a SNP unassociated with secondary phenotype and primary disease (MAF = 40%), at a 0.05 significance level in simulation studies using different approaches for HWP testing.
(DOC)
