# Supplementary material for: Testing Hardy-Weinberg Proportions in a Frequency-Matched Case-Control Genetic Association Study
Source: PLoS One. 2011 Nov 14;6(11):e27642. doi: 10.1371/journal.pone.0027642 (PMC3215743; doi:10.1371/journal.pone.0027642)
Supplement: Figure S1 — Construction of a mixture sample from the dataset of a case-control study of primary disease. (DOC) [file pone.0027642.s001.doc]

**Figure S1. Construction of a mixture sample from the dataset of a case-control study of primary disease**

*n* cases

*m* controls

*N_m_* mixture sample

$\hat{K}_{ij}$*:* Estimated joint probability of secondary phenotype status *i* and primary disease status *j*

Shaded circles and triangles: cases with respect to primary disease

White circles and triangles: controls with respect to primary disease

Circles: individuals with presence of secondary phenotype

Triangles: individuals with absence of secondary phenotype
