# Supplementary material for: Testing Hardy-Weinberg Proportions in a Frequency-Matched Case-Control Genetic Association Study
Source: PLoS One. 2011 Nov 14;6(11):e27642. doi: 10.1371/journal.pone.0027642 (PMC3215743; doi:10.1371/journal.pone.0027642)
Supplement: Figure S2 — Network structure representing associations among genetic variants, environmental factors, secondary phenotype, and primary disease. (DOC) [file pone.0027642.s002.doc]

**Figure S2. Network structure representing associations among genetic variants, environmental factors, secondary phenotype, and primary disease**
