# Supplementary material for: Testing Hardy-Weinberg Proportions in a Frequency-Matched Case-Control Genetic Association Study
Source: PLoS One. 2011 Nov 14;6(11):e27642. doi: 10.1371/journal.pone.0027642 (PMC3215743; doi:10.1371/journal.pone.0027642)
Supplement: Table S1 — Estimated type I error probability for test of deviation from HWP of SNP1, a SNP causal to both primary disease and secondary phenotype (MAF = 40%), at a 0.05 significance level in simulation studies using different approaches for HWP testing. (DOC) [file pone.0027642.s003.doc]

**Table S1. Estimated type I error probability for test of deviation from HWP of SNP_1_, a SNP causal to both primary disease and secondary phenotype (MAF = 40%), at a 0.05 significance level in simulation studies* using different approaches for HWP testing**

| **Approaches** | $f_{D}$ |  | $f_{T}$ | | | | | | | | |
| --- | --- | --- | --- | --- | --- | --- | --- | --- | --- | --- | --- |
|  |  |  | **0.1** | **0.2** | **0.3** | **0.4** | **0.5** | **0.6** | **0.7** | **0.8** | **0.9** |
|  |  |  |  |  |  |  |  |  |  |  |  |
| **LRT_t** | **0.1** |  | 0.370 | 0.342 | 0.311 | 0.335 | 0.308 | 0.321 | 0.337 | 0.342 | 0.350 |
|  | **0.2** |  | 0.234 | 0.229 | 0.210 | 0.194 | 0.200 | 0.207 | 0.217 | 0.251 | 0.220 |
|  | **0.3** |  | 0.130 | 0.131 | 0.092 | 0.107 | 0.107 | 0.123 | 0.126 | 0.142 | 0.143 |
|  | **0.4** |  | 0.066 | 0.072 | 0.086 | 0.066 | 0.051 | 0.058 | 0.066 | 0.077 | 0.066 |
|  | **0.5** |  | 0.042 | 0.047 | 0.051 | 0.042 | 0.045 | 0.049 | 0.043 | 0.057 | 0.047 |
|  | **0.6** |  | 0.078 | 0.067 | 0.059 | 0.068 | 0.079 | 0.076 | 0.068 | 0.070 | 0.070 |
|  | **0.7** |  | 0.124 | 0.138 | 0.122 | 0.116 | 0.122 | 0.117 | 0.115 | 0.111 | 0.122 |
|  | **0.8** |  | 0.238 | 0.215 | 0.220 | 0.213 | 0.225 | 0.202 | 0.193 | 0.212 | 0.197 |
|  | **0.9** |  | 0.369 | 0.343 | 0.334 | 0.341 | 0.330 | 0.295 | 0.326 | 0.348 | 0.358 |
|  |  |  |  |  |  |  |  |  |  |  |  |
| **mHWP_t** | **0.1** |  | 0.368 | 0.343 | 0.320 | 0.341 | 0.317 | 0.327 | 0.337 | 0.342 | 0.342 |
|  | **0.2** |  | 0.230 | 0.233 | 0.210 | 0.196 | 0.207 | 0.214 | 0.217 | 0.260 | 0.219 |
|  | **0.3** |  | 0.125 | 0.129 | 0.090 | 0.114 | 0.106 | 0.126 | 0.129 | 0.148 | 0.143 |
|  | **0.4** |  | 0.062 | 0.070 | 0.084 | 0.061 | 0.049 | 0.058 | 0.065 | 0.076 | 0.066 |
|  | **0.5** |  | 0.040 | 0.046 | 0.050 | 0.042 | 0.042 | 0.050 | 0.042 | 0.054 | 0.048 |
|  | **0.6** |  | 0.079 | 0.070 | 0.064 | 0.070 | 0.083 | 0.077 | 0.067 | 0.070 | 0.070 |
|  | **0.7** |  | 0.136 | 0.148 | 0.131 | 0.129 | 0.128 | 0.124 | 0.118 | 0.115 | 0.120 |
|  | **0.8** |  | 0.248 | 0.227 | 0.236 | 0.231 | 0.231 | 0.214 | 0.196 | 0.223 | 0.201 |
|  | **0.9** |  | 0.373 | 0.365 | 0.345 | 0.359 | 0.345 | 0.309 | 0.337 | 0.359 | 0.365 |
|  |  |  |  |  |  |  |  |  |  |  |  |
| **LRT_d** | **0.1** |  | 0.046 | 0.045 | 0.049 | 0.051 | 0.047 | 0.051 | 0.055 | 0.049 | 0.047 |
|  | **0.2** |  | 0.049 | 0.049 | 0.064 | 0.046 | 0.051 | 0.048 | 0.045 | 0.060 | 0.051 |
|  | **0.3** |  | 0.049 | 0.056 | 0.045 | 0.039 | 0.049 | 0.054 | 0.053 | 0.058 | 0.052 |
|  | **0.4** |  | 0.042 | 0.058 | 0.065 | 0.052 | 0.041 | 0.052 | 0.056 | 0.054 | 0.051 |
|  | **0.5** |  | 0.044 | 0.045 | 0.050 | 0.044 | 0.045 | 0.046 | 0.045 | 0.057 | 0.049 |
|  | **0.6** |  | 0.052 | 0.053 | 0.059 | 0.049 | 0.049 | 0.059 | 0.041 | 0.049 | 0.055 |
|  | **0.7** |  | 0.054 | 0.047 | 0.049 | 0.062 | 0.057 | 0.050 | 0.039 | 0.061 | 0.050 |
|  | **0.8** |  | 0.055 | 0.054 | 0.034 | 0.056 | 0.064 | 0.047 | 0.046 | 0.058 | 0.048 |
|  | **0.9** |  | 0.054 | 0.044 | 0.049 | 0.052 | 0.057 | 0.048 | 0.054 | 0.038 | 0.052 |
|  |  |  |  |  |  |  |  |  |  |  |  |
| **mHWP_d** | **0.1** |  | 0.052 | 0.045 | 0.052 | 0.057 | 0.047 | 0.055 | 0.055 | 0.051 | 0.050 |
|  | **0.2** |  | 0.055 | 0.051 | 0.063 | 0.049 | 0.053 | 0.052 | 0.049 | 0.061 | 0.053 |
|  | **0.3** |  | 0.056 | 0.060 | 0.048 | 0.041 | 0.049 | 0.057 | 0.055 | 0.059 | 0.053 |
|  | **0.4** |  | 0.046 | 0.060 | 0.068 | 0.057 | 0.047 | 0.058 | 0.057 | 0.061 | 0.056 |
|  | **0.5** |  | 0.041 | 0.045 | 0.047 | 0.043 | 0.044 | 0.044 | 0.043 | 0.054 | 0.042 |
|  | **0.6** |  | 0.055 | 0.055 | 0.063 | 0.053 | 0.055 | 0.063 | 0.044 | 0.052 | 0.057 |
|  | **0.7** |  | 0.061 | 0.056 | 0.049 | 0.067 | 0.058 | 0.050 | 0.041 | 0.065 | 0.052 |
|  | **0.8** |  | 0.060 | 0.060 | 0.036 | 0.061 | 0.063 | 0.049 | 0.048 | 0.060 | 0.049 |
|  | **0.9** |  | 0.054 | 0.046 | 0.050 | 0.056 | 0.058 | 0.048 | 0.056 | 0.037 | 0.051 |
|  |  |  |  |  |  |  |  |  |  |  |  |
| **eLRT** | **0.1** |  | 0.049 | 0.045 | 0.050 | 0.051 | 0.043 | 0.048 | 0.054 | 0.049 | 0.052 |
|  | **0.2** |  | 0.051 | 0.048 | 0.063 | 0.048 | 0.049 | 0.045 | 0.043 | 0.058 | 0.050 |
|  | **0.3** |  | 0.048 | 0.059 | 0.045 | 0.039 | 0.046 | 0.051 | 0.052 | 0.058 | 0.051 |
|  | **0.4** |  | 0.044 | 0.056 | 0.067 | 0.053 | 0.044 | 0.055 | 0.056 | 0.056 | 0.053 |
|  | **0.5** |  | 0.042 | 0.050 | 0.049 | 0.042 | 0.046 | 0.048 | 0.044 | 0.058 | 0.049 |
|  | **0.6** |  | 0.052 | 0.052 | 0.057 | 0.051 | 0.051 | 0.058 | 0.040 | 0.050 | 0.052 |
|  | **0.7** |  | 0.055 | 0.053 | 0.049 | 0.061 | 0.057 | 0.050 | 0.042 | 0.062 | 0.047 |
|  | **0.8** |  | 0.054 | 0.051 | 0.035 | 0.052 | 0.061 | 0.048 | 0.046 | 0.059 | 0.046 |
|  | **0.9** |  | 0.053 | 0.045 | 0.046 | 0.053 | 0.057 | 0.047 | 0.054 | 0.039 | 0.054 |
|  |  |  |  |  |  |  |  |  |  |  |  |
| **emHWP** | **0.1** |  | 0.048 | 0.047 | 0.051 | 0.054 | 0.049 | 0.053 | 0.060 | 0.050 | 0.049 |
|  | **0.2** |  | 0.054 | 0.051 | 0.070 | 0.048 | 0.050 | 0.047 | 0.051 | 0.062 | 0.049 |
|  | **0.3** |  | 0.053 | 0.063 | 0.046 | 0.041 | 0.047 | 0.055 | 0.050 | 0.064 | 0.057 |
|  | **0.4** |  | 0.043 | 0.058 | 0.068 | 0.056 | 0.046 | 0.060 | 0.059 | 0.060 | 0.056 |
|  | **0.5** |  | 0.044 | 0.049 | 0.052 | 0.043 | 0.044 | 0.049 | 0.044 | 0.058 | 0.048 |
|  | **0.6** |  | 0.057 | 0.055 | 0.063 | 0.053 | 0.054 | 0.064 | 0.042 | 0.052 | 0.056 |
|  | **0.7** |  | 0.058 | 0.054 | 0.052 | 0.065 | 0.059 | 0.052 | 0.046 | 0.066 | 0.050 |
|  | **0.8** |  | 0.055 | 0.053 | 0.036 | 0.054 | 0.062 | 0.054 | 0.048 | 0.062 | 0.048 |
|  | **0.9** |  | 0.052 | 0.050 | 0.049 | 0.057 | 0.056 | 0.048 | 0.057 | 0.036 | 0.055 |

*Simulation studies were based on 1,000 replicates, each replicate with 1,000 cases in terms of primary disease and 1,000 unmatched controls.

MAF: minor allele frequency

LRT_t: LRT approach, using presence and absence of secondary phenotype as cases and controls

mHWP_t: mHWP exact test, using presence and absence of secondary phenotype as cases and controls

LRT_d: LRT approach, using presence and absence of primary disease as cases and controls

mHWP_d: mHWP exact test, using presence and absence of primary disease as cases and controls

eLRT: extended LRT approach

emHWP: extended mHWP exact test

$f_{D}$: prevalence of primary disease in general population

$f_{T}$: prevalence of secondary phenotype in general population
