# Supplementary material for: Testing Hardy-Weinberg Proportions in a Frequency-Matched Case-Control Genetic Association Study
Source: PLoS One. 2011 Nov 14;6(11):e27642. doi: 10.1371/journal.pone.0027642 (PMC3215743; doi:10.1371/journal.pone.0027642)
Supplement: Table S2 — Estimated type I error probability for test of deviation from HWP of SNP2, a causal SNP to primary disease but unassociated with secondary phenotype (MAF = 40%), at a 0.05 significance level in simulation studies using different approaches for HWP testing. (DOC) [file pone.0027642.s004.doc]

**Table S2. Estimated type I error probability for test of deviation from HWP of SNP_2_, a causal SNP to primary disease but unassociated with secondary phenotype (MAF = 40%), at a 0.05 significance level in simulation studies* using different approaches for HWP testing**

| **Approaches** | $f_{D}$ |  | $f_{T}$ | | | | | | | | |
| --- | --- | --- | --- | --- | --- | --- | --- | --- | --- | --- | --- |
|  |  |  | **0.1** | **0.2** | **0.3** | **0.4** | **0.5** | **0.6** | **0.7** | **0.8** | **0.9** |
|  |  |  |  |  |  |  |  |  |  |  |  |
| **LRT_t** | **0.1** |  | 0.357 | 0.364 | 0.344 | 0.344 | 0.323 | 0.317 | 0.357 | 0.354 | 0.381 |
|  | **0.2** |  | 0.241 | 0.222 | 0.215 | 0.186 | 0.228 | 0.228 | 0.207 | 0.223 | 0.235 |
|  | **0.3** |  | 0.110 | 0.120 | 0.123 | 0.123 | 0.124 | 0.120 | 0.124 | 0.132 | 0.133 |
|  | **0.4** |  | 0.076 | 0.061 | 0.062 | 0.071 | 0.065 | 0.054 | 0.061 | 0.076 | 0.064 |
|  | **0.5** |  | 0.049 | 0.052 | 0.038 | 0.040 | 0.048 | 0.050 | 0.044 | 0.052 | 0.059 |
|  | **0.6** |  | 0.072 | 0.069 | 0.071 | 0.053 | 0.059 | 0.065 | 0.069 | 0.068 | 0.073 |
|  | **0.7** |  | 0.124 | 0.134 | 0.115 | 0.119 | 0.102 | 0.117 | 0.126 | 0.123 | 0.113 |
|  | **0.8** |  | 0.202 | 0.223 | 0.221 | 0.204 | 0.205 | 0.209 | 0.211 | 0.204 | 0.233 |
|  | **0.9** |  | 0.351 | 0.360 | 0.346 | 0.338 | 0.344 | 0.345 | 0.344 | 0.345 | 0.375 |
|  |  |  |  |  |  |  |  |  |  |  |  |
| **mHWP_t** | **0.1** |  | 0.359 | 0.362 | 0.348 | 0.355 | 0.331 | 0.332 | 0.360 | 0.352 | 0.378 |
|  | **0.2** |  | 0.235 | 0.224 | 0.215 | 0.190 | 0.234 | 0.234 | 0.219 | 0.229 | 0.234 |
|  | **0.3** |  | 0.110 | 0.119 | 0.122 | 0.124 | 0.127 | 0.121 | 0.129 | 0.137 | 0.131 |
|  | **0.4** |  | 0.073 | 0.057 | 0.060 | 0.069 | 0.063 | 0.056 | 0.062 | 0.074 | 0.067 |
|  | **0.5** |  | 0.048 | 0.052 | 0.039 | 0.041 | 0.046 | 0.047 | 0.042 | 0.051 | 0.061 |
|  | **0.6** |  | 0.074 | 0.071 | 0.072 | 0.056 | 0.063 | 0.067 | 0.069 | 0.069 | 0.069 |
|  | **0.7** |  | 0.134 | 0.141 | 0.122 | 0.125 | 0.104 | 0.122 | 0.130 | 0.127 | 0.111 |
|  | **0.8** |  | 0.207 | 0.228 | 0.241 | 0.223 | 0.224 | 0.217 | 0.220 | 0.213 | 0.238 |
|  | **0.9** |  | 0.356 | 0.369 | 0.361 | 0.358 | 0.365 | 0.366 | 0.359 | 0.360 | 0.382 |
|  |  |  |  |  |  |  |  |  |  |  |  |
| **LRT_d** | **0.1** |  | 0.061 | 0.057 | 0.057 | 0.038 | 0.039 | 0.055 | 0.046 | 0.058 | 0.059 |
|  | **0.2** |  | 0.055 | 0.069 | 0.047 | 0.043 | 0.046 | 0.046 | 0.053 | 0.045 | 0.064 |
|  | **0.3** |  | 0.047 | 0.048 | 0.047 | 0.044 | 0.045 | 0.060 | 0.047 | 0.052 | 0.050 |
|  | **0.4** |  | 0.053 | 0.040 | 0.054 | 0.059 | 0.047 | 0.036 | 0.050 | 0.054 | 0.037 |
|  | **0.5** |  | 0.050 | 0.054 | 0.039 | 0.040 | 0.050 | 0.049 | 0.046 | 0.052 | 0.062 |
|  | **0.6** |  | 0.047 | 0.052 | 0.058 | 0.037 | 0.052 | 0.048 | 0.050 | 0.039 | 0.045 |
|  | **0.7** |  | 0.048 | 0.042 | 0.047 | 0.039 | 0.040 | 0.047 | 0.053 | 0.048 | 0.038 |
|  | **0.8** |  | 0.043 | 0.045 | 0.052 | 0.041 | 0.051 | 0.054 | 0.046 | 0.048 | 0.054 |
|  | **0.9** |  | 0.046 | 0.051 | 0.048 | 0.056 | 0.031 | 0.057 | 0.050 | 0.050 | 0.045 |
|  |  |  |  |  |  |  |  |  |  |  |  |
| **mHWP_d** | **0.1** |  | 0.067 | 0.057 | 0.059 | 0.042 | 0.041 | 0.062 | 0.046 | 0.064 | 0.057 |
|  | **0.2** |  | 0.057 | 0.071 | 0.052 | 0.047 | 0.046 | 0.049 | 0.061 | 0.048 | 0.067 |
|  | **0.3** |  | 0.053 | 0.054 | 0.052 | 0.048 | 0.050 | 0.068 | 0.049 | 0.056 | 0.053 |
|  | **0.4** |  | 0.058 | 0.040 | 0.055 | 0.060 | 0.057 | 0.039 | 0.054 | 0.058 | 0.041 |
|  | **0.5** |  | 0.046 | 0.048 | 0.036 | 0.042 | 0.046 | 0.046 | 0.045 | 0.049 | 0.060 |
|  | **0.6** |  | 0.049 | 0.053 | 0.060 | 0.042 | 0.059 | 0.054 | 0.053 | 0.041 | 0.051 |
|  | **0.7** |  | 0.052 | 0.044 | 0.048 | 0.043 | 0.044 | 0.050 | 0.056 | 0.046 | 0.041 |
|  | **0.8** |  | 0.047 | 0.050 | 0.055 | 0.044 | 0.052 | 0.056 | 0.048 | 0.052 | 0.059 |
|  | **0.9** |  | 0.046 | 0.055 | 0.047 | 0.056 | 0.032 | 0.056 | 0.050 | 0.052 | 0.049 |
|  |  |  |  |  |  |  |  |  |  |  |  |
| **eLRT** | **0.1** |  | 0.060 | 0.057 | 0.058 | 0.038 | 0.041 | 0.054 | 0.045 | 0.061 | 0.056 |
|  | **0.2** |  | 0.054 | 0.066 | 0.046 | 0.042 | 0.043 | 0.046 | 0.054 | 0.044 | 0.062 |
|  | **0.3** |  | 0.047 | 0.049 | 0.045 | 0.041 | 0.045 | 0.058 | 0.045 | 0.051 | 0.048 |
|  | **0.4** |  | 0.051 | 0.039 | 0.054 | 0.059 | 0.048 | 0.036 | 0.051 | 0.054 | 0.036 |
|  | **0.5** |  | 0.050 | 0.054 | 0.039 | 0.043 | 0.049 | 0.048 | 0.044 | 0.056 | 0.058 |
|  | **0.6** |  | 0.046 | 0.052 | 0.058 | 0.037 | 0.051 | 0.048 | 0.052 | 0.039 | 0.048 |
|  | **0.7** |  | 0.047 | 0.043 | 0.046 | 0.042 | 0.040 | 0.048 | 0.053 | 0.048 | 0.038 |
|  | **0.8** |  | 0.045 | 0.047 | 0.053 | 0.042 | 0.053 | 0.054 | 0.045 | 0.048 | 0.054 |
|  | **0.9** |  | 0.047 | 0.049 | 0.051 | 0.059 | 0.031 | 0.055 | 0.051 | 0.049 | 0.049 |
|  |  |  |  |  |  |  |  |  |  |  |  |
| **emHWP** | **0.1** |  | 0.060 | 0.059 | 0.060 | 0.040 | 0.044 | 0.058 | 0.047 | 0.063 | 0.060 |
|  | **0.2** |  | 0.055 | 0.077 | 0.048 | 0.043 | 0.047 | 0.051 | 0.057 | 0.044 | 0.065 |
|  | **0.3** |  | 0.050 | 0.052 | 0.048 | 0.046 | 0.048 | 0.067 | 0.048 | 0.053 | 0.054 |
|  | **0.4** |  | 0.055 | 0.041 | 0.058 | 0.064 | 0.052 | 0.043 | 0.053 | 0.059 | 0.041 |
|  | **0.5** |  | 0.052 | 0.050 | 0.040 | 0.042 | 0.048 | 0.047 | 0.043 | 0.056 | 0.063 |
|  | **0.6** |  | 0.051 | 0.057 | 0.062 | 0.041 | 0.055 | 0.050 | 0.056 | 0.040 | 0.054 |
|  | **0.7** |  | 0.052 | 0.043 | 0.048 | 0.041 | 0.044 | 0.049 | 0.055 | 0.051 | 0.037 |
|  | **0.8** |  | 0.044 | 0.051 | 0.058 | 0.047 | 0.052 | 0.055 | 0.046 | 0.051 | 0.055 |
|  | **0.9** |  | 0.048 | 0.056 | 0.047 | 0.059 | 0.040 | 0.057 | 0.051 | 0.051 | 0.047 |

*Simulation studies were based on 1,000 replicates, each replicate with 1,000 cases in terms of primary disease and 1,000 unmatched controls.

MAF: minor allele frequency

LRT_t: LRT approach, using presence and absence of secondary phenotype as cases and controls

mHWP_t: mHWP exact test, using presence and absence of secondary phenotype as cases and controls

LRT_d: LRT approach, using presence and absence of primary disease as cases and controls

mHWP_d: mHWP exact test, using presence and absence of primary disease as cases and controls

eLRT: extended LRT approach

emHWP: extended mHWP exact test

$f_{D}$: prevalence of primary disease in general population

$f_{T}$: prevalence of secondary phenotype in general population
