# Supplementary material for: Testing Hardy-Weinberg Proportions in a Frequency-Matched Case-Control Genetic Association Study
Source: PLoS One. 2011 Nov 14;6(11):e27642. doi: 10.1371/journal.pone.0027642 (PMC3215743; doi:10.1371/journal.pone.0027642)
Supplement: Table S4 — Estimated type I error probability for test of deviation from HWP of SNP4, a SNP unassociated with secondary phenotype and primary disease (MAF = 40%), at a 0.05 significance level in simulation studies using different approaches for HWP testing. (DOC) [file pone.0027642.s006.doc]

**Table S4. Estimated type I error probability for test of deviation from HWP of SNP_4_, a SNP unassociated with secondary phenotype and primary disease (MAF = 40%), at a 0.05 significance level in simulation studies* using different approaches for HWP testing**

| **Approaches** | $f_{D}$ |  | $f_{T}$ | | | | | | | | |
| --- | --- | --- | --- | --- | --- | --- | --- | --- | --- | --- | --- |
|  |  |  | **0.1** | **0.2** | **0.3** | **0.4** | **0.5** | **0.6** | **0.7** | **0.8** | **0.9** |
|  |  |  |  |  |  |  |  |  |  |  |  |
| **LRT_t** | **0.1** |  | 0.050 | 0.054 | 0.048 | 0.046 | 0.038 | 0.050 | 0.049 | 0.054 | 0.050 |
|  | **0.2** |  | 0.046 | 0.041 | 0.052 | 0.042 | 0.049 | 0.059 | 0.048 | 0.056 | 0.047 |
|  | **0.3** |  | 0.039 | 0.064 | 0.059 | 0.050 | 0.062 | 0.060 | 0.049 | 0.048 | 0.056 |
|  | **0.4** |  | 0.050 | 0.048 | 0.064 | 0.059 | 0.058 | 0.043 | 0.060 | 0.057 | 0.054 |
|  | **0.5** |  | 0.045 | 0.038 | 0.044 | 0.053 | 0.050 | 0.057 | 0.039 | 0.053 | 0.058 |
|  | **0.6** |  | 0.048 | 0.048 | 0.049 | 0.053 | 0.054 | 0.051 | 0.051 | 0.037 | 0.050 |
|  | **0.7** |  | 0.047 | 0.050 | 0.055 | 0.064 | 0.052 | 0.050 | 0.041 | 0.057 | 0.036 |
|  | **0.8** |  | 0.049 | 0.050 | 0.053 | 0.063 | 0.049 | 0.052 | 0.045 | 0.052 | 0.049 |
|  | **0.9** |  | 0.042 | 0.044 | 0.063 | 0.052 | 0.056 | 0.040 | 0.051 | 0.046 | 0.056 |
|  |  |  |  |  |  |  |  |  |  |  |  |
| **mHWP_t** | **0.1** |  | 0.050 | 0.057 | 0.057 | 0.048 | 0.039 | 0.052 | 0.052 | 0.052 | 0.047 |
|  | **0.2** |  | 0.046 | 0.042 | 0.055 | 0.045 | 0.048 | 0.067 | 0.054 | 0.058 | 0.049 |
|  | **0.3** |  | 0.037 | 0.063 | 0.062 | 0.050 | 0.062 | 0.065 | 0.053 | 0.055 | 0.057 |
|  | **0.4** |  | 0.048 | 0.047 | 0.065 | 0.060 | 0.057 | 0.041 | 0.059 | 0.059 | 0.056 |
|  | **0.5** |  | 0.046 | 0.036 | 0.039 | 0.051 | 0.049 | 0.055 | 0.039 | 0.052 | 0.059 |
|  | **0.6** |  | 0.049 | 0.050 | 0.050 | 0.056 | 0.054 | 0.048 | 0.048 | 0.035 | 0.049 |
|  | **0.7** |  | 0.049 | 0.054 | 0.059 | 0.069 | 0.061 | 0.050 | 0.044 | 0.057 | 0.036 |
|  | **0.8** |  | 0.050 | 0.056 | 0.056 | 0.066 | 0.049 | 0.054 | 0.048 | 0.056 | 0.049 |
|  | **0.9** |  | 0.042 | 0.046 | 0.068 | 0.054 | 0.061 | 0.045 | 0.054 | 0.053 | 0.058 |
|  |  |  |  |  |  |  |  |  |  |  |  |
| **LRT_d** | **0.1** |  | 0.050 | 0.051 | 0.044 | 0.051 | 0.038 | 0.046 | 0.054 | 0.051 | 0.058 |
|  | **0.2** |  | 0.047 | 0.043 | 0.052 | 0.040 | 0.051 | 0.047 | 0.055 | 0.055 | 0.056 |
|  | **0.3** |  | 0.035 | 0.046 | 0.061 | 0.052 | 0.061 | 0.052 | 0.048 | 0.042 | 0.051 |
|  | **0.4** |  | 0.057 | 0.049 | 0.063 | 0.058 | 0.061 | 0.045 | 0.061 | 0.063 | 0.054 |
|  | **0.5** |  | 0.044 | 0.038 | 0.045 | 0.053 | 0.050 | 0.057 | 0.039 | 0.053 | 0.059 |
|  | **0.6** |  | 0.049 | 0.047 | 0.048 | 0.055 | 0.050 | 0.048 | 0.053 | 0.043 | 0.041 |
|  | **0.7** |  | 0.050 | 0.042 | 0.048 | 0.065 | 0.061 | 0.046 | 0.043 | 0.059 | 0.038 |
|  | **0.8** |  | 0.056 | 0.053 | 0.051 | 0.054 | 0.065 | 0.046 | 0.031 | 0.052 | 0.041 |
|  | **0.9** |  | 0.051 | 0.040 | 0.057 | 0.043 | 0.056 | 0.045 | 0.048 | 0.046 | 0.045 |
|  |  |  |  |  |  |  |  |  |  |  |  |
| **mHWP_d** | **0.1** |  | 0.050 | 0.050 | 0.046 | 0.051 | 0.039 | 0.047 | 0.058 | 0.055 | 0.062 |
|  | **0.2** |  | 0.048 | 0.049 | 0.063 | 0.041 | 0.056 | 0.049 | 0.060 | 0.061 | 0.057 |
|  | **0.3** |  | 0.040 | 0.050 | 0.064 | 0.056 | 0.064 | 0.057 | 0.051 | 0.048 | 0.054 |
|  | **0.4** |  | 0.062 | 0.052 | 0.063 | 0.065 | 0.065 | 0.051 | 0.067 | 0.065 | 0.064 |
|  | **0.5** |  | 0.043 | 0.035 | 0.038 | 0.047 | 0.048 | 0.055 | 0.038 | 0.046 | 0.057 |
|  | **0.6** |  | 0.054 | 0.054 | 0.052 | 0.060 | 0.051 | 0.055 | 0.057 | 0.045 | 0.042 |
|  | **0.7** |  | 0.056 | 0.048 | 0.050 | 0.068 | 0.067 | 0.049 | 0.048 | 0.057 | 0.045 |
|  | **0.8** |  | 0.060 | 0.055 | 0.055 | 0.059 | 0.069 | 0.049 | 0.031 | 0.059 | 0.045 |
|  | **0.9** |  | 0.049 | 0.044 | 0.056 | 0.046 | 0.059 | 0.046 | 0.052 | 0.052 | 0.047 |
|  |  |  |  |  |  |  |  |  |  |  |  |
| **eLRT** | **0.1** |  | 0.048 | 0.049 | 0.043 | 0.051 | 0.037 | 0.047 | 0.053 | 0.053 | 0.055 |
|  | **0.2** |  | 0.048 | 0.044 | 0.055 | 0.040 | 0.053 | 0.046 | 0.056 | 0.059 | 0.053 |
|  | **0.3** |  | 0.035 | 0.046 | 0.061 | 0.051 | 0.062 | 0.053 | 0.048 | 0.043 | 0.049 |
|  | **0.4** |  | 0.056 | 0.049 | 0.060 | 0.056 | 0.065 | 0.048 | 0.060 | 0.062 | 0.051 |
|  | **0.5** |  | 0.045 | 0.037 | 0.044 | 0.054 | 0.050 | 0.056 | 0.039 | 0.053 | 0.057 |
|  | **0.6** |  | 0.050 | 0.047 | 0.047 | 0.055 | 0.052 | 0.050 | 0.051 | 0.042 | 0.044 |
|  | **0.7** |  | 0.048 | 0.042 | 0.050 | 0.066 | 0.060 | 0.047 | 0.041 | 0.058 | 0.038 |
|  | **0.8** |  | 0.061 | 0.052 | 0.050 | 0.057 | 0.064 | 0.045 | 0.031 | 0.052 | 0.043 |
|  | **0.9** |  | 0.045 | 0.043 | 0.057 | 0.043 | 0.048 | 0.045 | 0.047 | 0.048 | 0.045 |
|  |  |  |  |  |  |  |  |  |  |  |  |
| **emHWP** | **0.1** |  | 0.056 | 0.053 | 0.048 | 0.051 | 0.041 | 0.051 | 0.060 | 0.056 | 0.063 |
|  | **0.2** |  | 0.046 | 0.047 | 0.058 | 0.042 | 0.056 | 0.049 | 0.060 | 0.064 | 0.057 |
|  | **0.3** |  | 0.040 | 0.053 | 0.063 | 0.054 | 0.065 | 0.054 | 0.048 | 0.048 | 0.054 |
|  | **0.4** |  | 0.061 | 0.053 | 0.066 | 0.065 | 0.071 | 0.054 | 0.063 | 0.065 | 0.060 |
|  | **0.5** |  | 0.046 | 0.039 | 0.044 | 0.053 | 0.051 | 0.056 | 0.040 | 0.052 | 0.061 |
|  | **0.6** |  | 0.053 | 0.054 | 0.054 | 0.057 | 0.054 | 0.054 | 0.056 | 0.045 | 0.045 |
|  | **0.7** |  | 0.052 | 0.047 | 0.050 | 0.070 | 0.065 | 0.050 | 0.047 | 0.058 | 0.041 |
|  | **0.8** |  | 0.063 | 0.052 | 0.058 | 0.058 | 0.068 | 0.049 | 0.034 | 0.056 | 0.042 |
|  | **0.9** |  | 0.054 | 0.047 | 0.062 | 0.046 | 0.060 | 0.049 | 0.051 | 0.049 | 0.046 |

*Simulation studies were based on 1,000 replicates, each replicate with 1,000 cases in terms of primary disease and 1,000 unmatched controls.

MAF: minor allele frequency

LRT_t: LRT approach, using presence and absence of secondary phenotype as cases and controls

mHWP_t: mHWP exact test, using presence and absence of secondary phenotype as cases and controls

LRT_d: LRT approach, using presence and absence of primary disease as cases and controls

mHWP_d: mHWP exact test, using presence and absence of primary disease as cases and controls

eLRT: extended LRT approach

emHWP: extended mHWP exact test

$f_{D}$: prevalence of primary disease in general population

$f_{T}$: prevalence of secondary phenotype in general population
